# Egyptian evidence-based consensus on clinical practice guidelines for the diagnosis and treat-to-target management of macrophage activation syndrome in children

**DOI:** 10.1186/s43166-022-00135-z

**Published:** 2022-06-13

**Authors:** H. Lotfy, M. H. Abu-Zaid, S. Salah, M. El Gaafary, H. Abdulhady, H. Salah, E. Abd El-Latif, Y. Farag, M. Eissa, S. Esam Maher, A. Radwan, Amira T. El-Shanawany, B. M. Medhat, D. El Mikkawy, D. M. Mosa, G. El Deriny, M. Mortada, N. S. Osman, N. A. Fouad, N. E. Elkaraly, S. S. Mohamed, S. A. Tabra, W. A. Hassan, H. Abu Shady, Y. Amer, S. I. Nasef, Salwa Galal, Y. El Miedany

**Affiliations:** 1 Cairo University, Cairo, Egypt; 2 Rheumatology and Rehabilitation Department, Faculty of Medicine, Tanta University, El-Geish Street, Tanta, Gharbia 31527 Egypt; 3 Ain Shams University, Cairo, Egypt; 4 Alexandria University, Alexandria, Egypt; 5 Minia University, Minia, Egypt; 6 Sohag University, Sohag, Egypt; 7 Menoufia University, Menoufia, Egypt; 8 Mansoura University, Mansoura, Egypt; 9 Zagazig University, Zagazig, Egypt; 10 Assiut University, Assiut, Egypt; 11 Fayoum University, Fayoum, Egypt; 12 Suez Canal University, Ismailia, Egypt; 13 Benha University, Benha, Egypt; 14 Canterbury Christ Church University, Kent, England; 15 King’s College London, London, England

**Keywords:** Macrophage activation syndrome, Treat to target, Egyptian guidelines MAS, Algorithm

## Abstract

**Background:**

Macrophage activation syndrome (MAS) is a severe life-threatening hyperinflammatory state with uncontrolled activation and proliferation of macrophages and T-lymphocytes. MAS has variable causes and risk factors. Early diagnosis and optimum management could be lifesaving.

Our aim was to develop a consensus, evidence-based recommendations for the diagnosis, evaluation, and treat-to-target management of pediatric MAS.

This study was carried out to achieve an Egyptian expert consensus on a treat-to-target management strategy for MAS using the Delphi technique. The multistep process strategy was used in developing a consensus, evidence-based treatment guidelines for MAS, started by developing 7 key clinical questions by a scientific committee according to the Patient/Population, Intervention, Comparison, and Outcomes (PICO) approach. The core leadership team identified pediatric rheumatology clinicians and researchers throughout Egypt. To generate evidence for MAS management, an evidence-based, systematic literature review was done. To obtain a consensus, the Delphi procedure (3 rounds) was used.

**Results:**

Twenty-three expert panel participated in the 3 rounds with a response rate of 100%. A total of 19 recommendations, categorized into 2 sections (11 in the diagnosis section and 8 in management), were obtained. The agreement with the recommendations (ranks 7–9) ranged from 86.9 to 95.7%. The consensus was reached (i.e., ≥75% of respondents strongly agreed or agreed) on all the clinical standards. Algorithms for management have been also developed.

**Conclusion:**

This was an expert, consensus recommendation for the diagnosis and treat to target of MAS, based on the best available evidence and expert opinion. The guidelines fill a gap in the literature as it presents a T2T approach for MAS.

## Background

Macrophage activation syndrome (MAS) is also considered a type of secondary hemophagocytic lymphohistiocytosis (sHLH) associated with rheumatological diseases [1]. MAS is characterized by uncontrolled macrophage and T-lymphocyte activation and proliferation, as well as a significant increase in circulating cytokines such as IFN-gamma and GM-CSF [2]. Furthermore, its pathophysiology is still unknown, and various factors, including genetic factors, may play a role in the etiology and pathogenesis of MAS [3] demonstrating the link between MAS risk and interleukin-18. The most prevalent clinical signs of MAS include prolonged high fever, hepatosplenomegaly, neurologic impairment, and hemorrhagic abnormalities. Pancytopenia, high serum liver enzymes, triglycerides, lactate dehydrogenase, and ferritin, as well as low fibrinogen levels, are all common laboratory findings. Despite the fact that macrophage hemophagocytosis is a common finding in bone marrow examinations, this finding may be missed in the early stages of MAS [4, 5]. The exact incidence of MAS is still unknown because of the wide range of clinical signs and the possibility of sustaining episodes of the disease that may pass unnoticed clinically [2].

Similar to its pathogenesis, treatment of MAS has been a matter of controversy. Parenteral treatment of high doses of corticosteroids has typically been used to treat MAS; nonetheless, occasional fatalities have been seen even among patients receiving high doses of corticosteroids. High-dose intravenous immunoglobulins, cyclophosphamide, plasma exchange, and etoposide have all been used with conflicting results [6–8].

Despite the fact that early diagnosis is associated with better outcomes, early detection of MAS remains a challenge. This has been attributed to the lack of a single pathognomonic trait or even a set of universal diagnostic criteria for MAS. Similarly, early management is a true life-saving competition [9]. There are few international guidelines for the management of MAS; the most recent, the Classification Criteria for Macrophage Activation Syndrome Complicating Systemic Juvenile Idiopathic Arthritis, released in 2016, focused solely on diagnosis rather than treatment. Furthermore, there are currently no Egyptian-wide, evidence-based, treat-to-target recommendations for the proper diagnosis and treatment of MAS in children. This was the motivating force for the development of this work. The objective is to provide a consensus, evidence-based recommendations for the diagnosis, evaluation, and treat-to-target management of children living with MAS. Although these guidelines were created for Egyptian children with MAS, we hope that they would be helpful to pediatric rheumatologists all around the world.

## Methods

### Design

The study design was developed using scientific evidence and consensus, which was based on both existing scientific evidence and clinical experience. The purpose of this multistep method was to develop a clinical gold standard for MAS treat-to-target management in children using the “Clinical, Evidence-based, Guidelines” (CEG) initiative protocol (ethical approval number: 34842/8/21). The evidence-based component of the manuscript adhered to the preferred reporting items for systematic reviews and meta-analyses criteria for systematic review publication [10]. The Egyptian College of Pediatric Rheumatology was the driving force behind the project.

### Development stages

#### Core team

It is made up of four professionals with backgrounds in pediatric rheumatology, inflammatory arthritis, and macrophage activation syndrome. The core team was involved in managing and coordinated the work of the team; assisted in the development of the project’s scope and initial Patient/Population, Intervention, Comparison, and Outcomes (PICO) clinical questions; came to an agreement on the key questions to be included in the guidelines; nominated the expert panel; and drafted the manuscript.

#### Key questions used to develop the guidelines

The target population, categorization criteria, the intervention, diagnostic test, or exposure under investigation, the comparison(s), and the outcomes used to quantify efficacy, effectiveness, or risk were all defined by a set of structured key questions. The following methods were used to collect evidence to answer the clinical questions: formulation of clinical questions, structure of questions, search for evidence, critical evaluation and selection of evidence, presentation of results, and recommendations. The systematic literature search and, as a result, clinical care standards are based on these questions, as indicated in Table 1.

**Table 1:** Key questions used to develop the guideline

1- Early diagnostic tools	
2- Diagnostic criteria of MAS with sJIA	
3- MAS presentations with different diseases	
- Diagnostic criteria for MAS complicating systemic lupus erythematosus	
- Systemic auto-inflammation	
- Kawasaki disease	
4. Differential diagnosis	
- sJIA activity	
- Systemic auto-inflammatory diseases (SAIDS)	
- Kawasaki disease	
- Lupus	
- Infection	
5. Treat-to-target strategy	
4- Management	
- Prevention	
- First line of management	
- Monitoring and follow-up (clinical and laboratory)	
5- Define resistant and irresponsive/severe cases of MAS	
6- Management of resistant and severe cases	
7- MAS and COVID-19	

*MAS* macrophage activation syndrome, *sJIA* systemic onset juvenile idiopathic arthritis, *SAIDS* systemic auto-inflammatory diseases, *COVID-19* coronavirus disease of 2019

#### Literature review team

The literature review was carried out with the help of a methodology expert, under the supervision of an experienced literature review consultant, and was based on specific research topics relating to the diagnosis and treatment of MAS. The team finished the literature search (using the PubMed/MEDLINE, EMBASE, and Cochrane databases), data abstraction, and evidence quality evaluation [11]. Following the revision, each of the literature review experts made recommendations for each section based on evidence or their own personal experience. The Oxford Centre for Evidence-based Medicine (CEBM) approach was used to establish the degree of evidence for each section [12].

#### Data sources and search strategies

The PICO questions (Table 1) were used to conduct the literature search. On August 10, 2021, the first systematic literature search was undertaken, which included all English publications published since 2000 in the EMBASE, PubMed, and Cochrane databases. The keywords that were used were determined by the PICO elements that were used in various combinations. Literature searches on 10 March 2021 for PubMed and Cochrane Library databases and on 28 March 2021 for EMBASE. PubMed review was carried out by searching for the medical subject headings (MeSH) (Lymphohistiocytosis, Hemophagocytic) and (Macrophage Activation Syndrome, (arthritis, Juvenile arthritis) supplemented with the keywords MAS, lymphohistiocytosis, JIA, sJIA, jSLE, Kawasaki disease, infectious diseases, Still’s disease, and synonyms. For EMBASE, MeSH terms were replaced by the corresponding Emtree terms. The Cochrane Central Register of Controlled Trials (CENTRAL) was searched for the same keywords. On April 25, 2021, the search was updated to include the most recently released publications. Electronically, duplicate screening of literature search results was performed. Additional papers that satisfied the inclusion criteria were found by looking through the reference lists of studies found using database search tools.

#### Study selection

Relevant studies were chosen using inclusion and exclusion criteria applied to the literature found using the search methodology.

#### Inclusion criteria

Articles included were systematic reviews, randomized controlled trials (RCTs), uncontrolled trials, and observational studies including cohort, case-control, and cross-sectional studies.

#### Exclusion criteria

Conference abstracts, commentaries, editorials, and non-evidence-based personal/narrative reviews were excluded.

#### Expert panel

The core leadership team nominated 23 participants. The criteria for their selection included practice in the Egyptian Health System, professional knowledge, and experience in the field of pediatric rheumatology (minimum 8 years) especially including management of inflammatory arthritis and macrophage activation syndrome. The expert panel contributed in creating the scope of the project, refining the PICO questions, and voting on the recommendations by actively participating in scientific research on pediatric rheumatic disorders.

### Developing the clinical care standards framework

To aid in the standardized identification of the guideline components, a structured template was created based on the answers to the structured key questions and the literature research. For each guideline component, the format in which the recommendations/information will be presented and extracted has been determined.

#### Delphi process

The Delphi method is a structured method for gathering vital information about a certain issue that is extensively used. It is predicated on the notion that group projections are more accurate than individual forecasts. The Delphi method’s goal is to build consensus forecasts from a group of experts in a structured iterative manner. Its methodology is based on a succession of “rounds” of questionnaires sent to experts. The Delphi method generally involves the following stages: (1) A group of experts is gathered together. (2) Forecasting tasks/challenges are assigned to professionals and distributed. (3) Experts provide preliminary forecasts and justifications. In order to provide feedback, these are collated and summarized. (4) The experts receive comments, which they consider when revising their forecasts. This process can be repeated until there is a reasonable level of consensus. (5) The final forecasts are created by combining the forecasts of the experts. The key features of this method are the anonymity of participants and the controlled feedback [13–15].

#### Consensus process

To reach a consensus on the T2T (treat to target) strategy in MAS, three Delphi rounds were conducted. After the major features of the approach were determined, a discussion group worked with the scientific committee to specify the aspects that would be included in the questionnaire. The structured Delphi approach ensures that all participants’ perspectives are taken into account equally, and it is especially effective for geographically diverse cities like Egypt. Online surveys were used to conduct the Delphi procedure. The Internet questionnaire’s first round featured 20 issues related to MAS’s T2T strategy.

#### Voting process

Three rounds of live online voting were held, each with a strict time limit. All members of the task force were invited to participate, and the start and end times of each round of voting were announced ahead of time. Anonymous votes were gathered and evaluated, and we handed out the unique access links. At the same time as the voting procedure, comments on rephrasing, potential ambiguity, and unidentified overlaps were received for each statement. The task force members were the only ones who could vote on the statements.

#### Rating

Each statement was rated between 1 and 9 with 1 being “complete disagreement” and 9 being “complete agreement.” Generally, 1–3, 4–6, and 7–9 represent disagreement, uncertainty, and agreement, respectively. It is not necessary for members to vote on all statements, and they are invited to abstain if they believe a statement is outside their area of competence. As a result, a vote of “uncertainty” indicates “inconvenience about the accuracy of the recommendation.” All statements allow for the submission of comments, which the scientific committee reviews after each round of voting. Members were also encouraged to make remarks wherever they voted a disagreement in all of the voting rounds. This will allow the panel to spot a case of statement misinterpretation and nullify the vote on that statement.

#### Definition of consensus

Prior to data analysis, a definition of consensus was defined. It was concluded that if at least 75% of the participants agreed (scoring 7–9) or disagreed (scores 1–3), consensus would be attained [11–14]. If a statement received a mean vote of less than 3 or a “poor” degree of agreement, it was retired. In light of the feedback, statements with an uncertainty score of 4–6 were changed. Following the second round of voting, the levels of agreement on each statement of recommendation were rated “strong” if all votes on that statement fell into the agreement bracket (7–9) [15–17].

#### Chronogram of Delphi rounds

The first round took place between 12 and 15 May 2021 (4 days). The aspects about which respondents did not reach consensus in this first round were revised in view of the comments and included in the second round. The second round took place (1 week after the first round) and remained for 4 days, between 22 and 25 May 2021 (4 days). The third round took place (2 weeks after the second round) and remained for 4 days between 11 and 14 June 2021 (4 days).

### Ethical aspects

This study was performed in accordance with the Helsinki Declaration. The “Clinical, Evidence-based, Guidelines” (CEG) initiative protocol was approved by the local ethical committee: ethical approval code: 34842/8/21, ethical board Tanta University. According to national standards, written ethics approval from the experts involved in this project was not required. In accordance with data protection standards, all participants were kept anonymous.

## Results

### Literature research and evidence selection

**Fig. 1:**
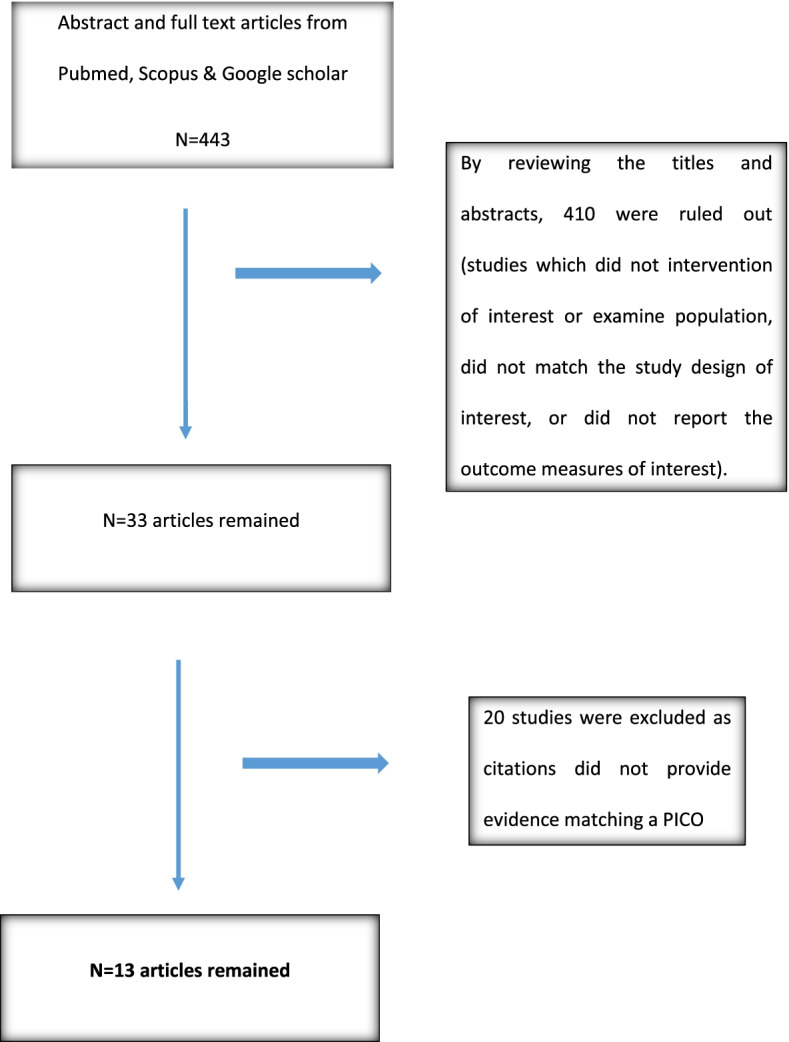
Flow chart for the study selection process

In the study selection process, we found 443 potentially relevant studies by search strategy. In total, 410 were excluded by screening of title and abstracts (for duplication or the studies did not examine population or intervention of interest, did not match study design of interest, or did not report outcome measures of interest). Therefore, relevant 33 studies were included for full article review. Twenty studies were excluded as citations did not provide evidence matching a PICO. Therefore, we included 13 studies in this work (Fig. 1).

### Expert panel characteristics

The Delphi form was sent to the expert panel (*n* = 23), who participated in the three rounds. Respondents were drawn from different governorates and health centers across Egypt: Cairo University (26.1%), Ain Shams University (13.05%), Tanta University (8.7%), Benha University (4.35%), Alexandria University (4.35%), Suez Canal University (8.7%), Zagazig University (8.7%), Minia University (4.35%), Mansoura University (4.35%), Fayoum University (4.35%), Assiut University (4.35%), Menofeya University (4.35%), and Sohag University (4.35%).

The mean years of experience in the field of pediatric rheumatology of the expert panel were 25.1, Cairo University; 23.55, Ain Shams University; 14.5, Tanta University; 18, Benha University; 18, Alexandria University; 13, Suez Canal University; 18, Zagazig University; 25, Minia University; 15, Mansoura University; 18, Fayoum University; 13, Assiut University; 10, Menofeya University; and 18, Sohag University.

### Delphi round 1 (MAS guidelines clinical questions) (Table 1)

In this phase, we introduced the clinical questions which will be the base and the titles of items in this guidelines work. The response rate for round 1 was 100% (23/23). Consensus was reached on the inclusion of clinical standards on 90% of the items (i.e., ≥ 75% of respondents strongly agreed or agreed). There were comments raised regarding the wording of some of the recommendations. MAS presentations with various disorders, as well as MAS and COVID-19, received more comments (excluding minor editing suggestions). The number of statements which were added after round 1 was 1 in the diagnostic criteria section, 2 in the management section, and 2 statements in the MAS and COVID-19 section.

### Delphi round 2

The response rate for round 2 was 100% (23/23). Consensus was reached on the inclusion of clinical standards on 89.6% of the items (i.e., ≥ 75% of respondents strongly agreed or agreed). There were comments raised regarding the wording of some of the recommendations. Comments (excluding minor editing suggestions) were more frequent for MAS presentations with different diseases, differential diagnosis, monitoring and follow-up, and management of resistant and severe cases section. Diversity of opinion was greatest for the item “management of resistant and severe cases.” The number of statements which were added after round 2 was one statement in the differential diagnosis section, three statements were added to the monitoring and follow-up section, and two statements were added to the MAS and COVID-19 section. Several statements were revised after round 2; most edited statements were in the management of resistant and severe cases section (5 statements), one statement was amended in each of key point, early diagnostic tools, treat-to-target strategy, and MAS and COVID-19 sections.

### Delphi round 3

The response rate for round 3 was 100% (23/23). The frequency of high rank recommendation (ranks 7–9) ranged between 86.9 and 95.7%. One statement was retired for similarity with another statement. Consensus was reached (i.e., ≥75% of respondents strongly agreed or agreed) on all the clinical standards. Table 2 shows the level of evidence assigned to each statement, in accordance with the Oxford Centre for Evidence-Based Medicine (CEBM) criteria as well as mean ± standard deviation and level of agreement [11]. Agreement was unanimous (>80% agreement) for the wording of the statements.

**Table 2:** Levels of evidence

**Level of evidence**	
1	Systematic review of all relevant randomized clinical trials or n-of-1 trials
2	Randomized trial or observational study with dramatic effect
3	Non-randomized controlled cohort/follow-up study (observational)
4	Case series, case-control study, or historically controlled study
5	Mechanism-based reasoning (expert opinion, based on physiology, animal or laboratory studies)
**Grades of recommendation**	
A	Consistent level 1 studies
B	Consistent level 2 or 3 studies, or extrapolations from level 1 studies
C	Level 4 studies, or extrapolations from level 2 or 3 studies
D	Level 5 evidence or troubling, inconsistent, or inconclusive studies of any level

### Recommendations for the management of children with MAS

At the end of Delphi round 3, a total of nineteen recommendation items were concluded. These were categorized into 2 sections: 11 recommendations under the diagnosis and 8 under management. Table 3 presents the 2016 Classification Criteria for Macrophage Activation Syndrome complicating Systemic Juvenile Idiopathic Arthritis, which the patients should fulfill to be classified into MAS, though patient management should be commenced as soon as possible, even if the diagnosis of MAS is suspected, as this is a life-threatening condition.

**Table 3:** 2016 classification criteria for macrophage activation syndrome (MAS) complicating systemic juvenile idiopathic arthritis [18]

A febrile patient with known or suspected systemic juvenile idiopathic arthritis is classified as having macrophage activation syndrome if the following criteria are met:	
- Ferritin >684 ng/mL and any two of the following:	
° Platelet count ≤ 181 × 10^9^/L	
° Aspartate aminotransferase (AST) > 48 units/L	
° Triglycerides > 156 mg/dL	
° Fibrinogen ≤ 360 mg/dL	

*AST* aspartate aminotransferase, *MAS* macrophage activation syndrome

### Early diagnostic tools

There was a high level of agreement 8.26 + 1.7 (agreement percentage: 95.65%) on the criteria for early diagnosis of MAS (level of evidence (LE) 3, strength of recommendation (SoR) B, level of agreement H). These tools are summarized in Table 4.

**Table 4:** Early diagnostic tools in MAS complicating sJIA

Statement	LE	SoR	Mean rate± SD	% of agreement	Level of agreement
- Progressive increase in serum ferritin is a valuable laboratory marker showing the largest change in both pre-MAS and MAS-onset values (often > 10,000 ng/mL) - Dropping ESR levels helps to distinguish MAS from a flare of the underlying rheumatic disorder (where ESR is usually elevate (a drop in ESR or a disproportion between ESR and CRP levels would raise the suspicion of MAS)) - As some patients may have basic elevated serum ferritin levels especially with repeated blood transfusions, a progressive increase in serum ferritin from the basic level for these patients would be suggestive of MAS particularly if it was associated with a decrease of ESR - Relative decrease in platelet count followed by a decrease in WBCs, or fibrinogen levels rather than an absolute decrease, may be more useful in making an early diagnosis of MAS and differentiate MAS from sJIA flare - Hemophagocytosis in bone marrow examination is pathognomonic, but failure to reveal hemophagocytosis does not exclude the diagnosis of MAS as histopathologic features of hemophagocytosis may not be present in the initial stages	3	B	8.26 ± 1.7	95.65	H

*LE* level of evidence according to the Oxford Centre for Evidence-Based Medicine (CEBM) criteria, *H* high level of agreement, *SoR* strength of recommendation, *MAS* macrophage activation syndrome, *sJIA* systemic onset juvenile idiopathic arthritis, *ESR* erythrocyte sedimentation rate, *CRP* C-reactive protein

As MAS could complicate many disorders, MAS presentations with different diseases have been assessed and presented in Table 5; also, Table 5 shows an approach to differentiate between MAS and disease activities.

**Table 5:** MAS presentations in different diseases and differentiation between MAS and disease activities

Standard	Statement	LE	SoR	Mean rate ± SD	% of agreement	Level of agreement
**MAS presentations with different diseases**						
***SLE***	**- Diagnostic criteria for macrophage activation syndrome complicating systemic lupus erythematosus*(preliminary diagnostic guidelines for MAS as a complication of juvenile SLE according to Parodi et al.)*** [19] The diagnosis of MAS requires the simultaneous presence of at least 1 clinical criterion and at least 2 laboratory criteria. Bone marrow aspiration for evidence of macrophage hemophagocytosis may be required only in doubtful cases. *Clinical criteria* 1. Fever (>38°C) 2. Hepatomegaly (>3 cm below the costal arch) 3. Splenomegaly (>3 cm below the costal arch) 4. Hemorrhagic manifestations (purpura, easy bruising, or mucosal bleeding) 5. Central nervous system dysfunction (irritability, disorientation, lethargy, headache, seizures, or coma) *Laboratory criteria* 1. Cytopenia affecting 2 or more cell lineages (white blood cell count ≤ 4.0 × 10^9^/L, hemoglobin ≤ 90 gm/L, or platelet count ≤ 150 × 10^9^/L 2. Increased aspartate aminotransferase >40 units/L) 3. Increased lactate dehydrogenase (>567 units/L) 4. Hypofibrinogenemia (fibrinogen ≤1.5 gm/L) 5. Hypertriglyceridemia (triglycerides >178 mg/dL) 6. Hyperferritinemia (ferritin >500 μg/L) *Histopathologic criterion* Evidence of macrophage hemophagocytosis in the bone marrow aspirate	3	B	8.3 ± 1.4	91.3	H
***Systemic auto-inflammation***	- *Clinically*: persistent fever, fatigue, hepatosplenomegaly, hepatic impairment, serositis, lymphadenopathy, case response to corticosteroid treatment - *Lab*: dropping of platelets (disproportionate with other inflammatory markers), pancytopenia, low fibrinogen level, clotting abnormalities, hyponatremia, perforin gene mutation, evidence of macrophage hemophagocytosis in the bone marrow aspirate	3	B	8.04 ± 1.8	91.3	H
**Kawasaki disease**	- MAS may occur in any stage of KD (acute stage, subacute stage, or recovery stage) and may also occur prior to a KD diagnosis, but in most cases, it appears simultaneously with KD - Hepatosplenomegaly, neurological manifestations - Cytopenia, drop in ESR, or a disproportion between ESR and CRP levels, hyperferritinemia - Though bone marrow evidence of hemophagocytosis can be pathognomonic, failure to reveal hemophagocytosis does not exclude the diagnosis of MAS as histopathologic features of hemophagocytosis may not be present in the initial stages	3	B	7.86 ± 1.9	95.65	H
**Differentiation between MAS and disease activities**						
***sJIA***	- Usually, MAS occurred in clinically active and resistive cases - MAS develops in the earlier phases or may be the presenting manifestation of sJIA; however, onset has been reported as long as 14 years after the initial diagnosis - The child complains of fatigue, tiredness, persistent high fever, more prominent hepatosplenomegaly, and lymphadenopathy, and rashes become petechial not evanescent salmon-pink rashes - MAS-associated SJIA tends to have more hepatosplenomegaly and lymphadenopathy than does MAS-associated SLE - Platelet count drop is the first most common early manifestations of MAS in sJIA patients, pancytopenia, dropped ESR due to hypofibrinogenemia, disproportion between ESR and CRP levels - Serum ferritin levels are the highest in the MAS-associated SJIA condition - So, persistent fever, neurological manifestations, and dropping of ESR are the most differentiating features between sJIA and MAS	1	A	8.34 ± 1.8	95.65	H
***Systemic auto-inflammatory diseases (SAIDS)***	- When MAS complicating SAIDS: - Fever becomes persistent, unremitting, high-grade, apparently unexplained - Rashes become petechial not polymorphic - Serositis becomes more severe, with prominent hepatosplenomegally - Neurological symptoms may occur - Pancytopenia, elevated serum transaminases, hypofibrinogenemia - In contrast in SAIDS, high ESR, procalcitonin, and CRP are usually reported in SAIDS	2	B	8.26 ± 1.8	91.3	H
**Kawasaki disease**	- - MAS-associated Kawasaki (KD) patients always present hepatosplenomegaly, whereas this is an uncommon presentation in patients with active KD	3	B	8.09 ± 2.3	88.9	H
**SLE**	- MAS may occur in lupus patients, sometimes as the first presentation, although not as common as sJIA - Lab tests in favor of SLE flare include nephritis, hypocomplementemia, and elevated ESR	2	B	8.3 ± 1.8	88.9	H
**Infection**	- Fever becomes more persistent not responding to antipyretics, with poor general status Hepatosplenomegaly, lymphadenopathy, petechial rashes, and neurological insult may occur Pancytopenia, dropped ESR due to hypofibrinogenemia, disproportion between ESR and CRP	2	B	8.17 ± 1.8	88.9	H

*LE* level of evidence according to the Oxford Centre for Evidence-Based Medicine (CEBM) criteria, *H* high level of agreement, *SoR* strength of recommendations, *MAS* macrophage activation syndrome, *sJIA* systemic onset juvenile idiopathic arthritis, *KD* Kawasaki disease

**Fig. 2:**
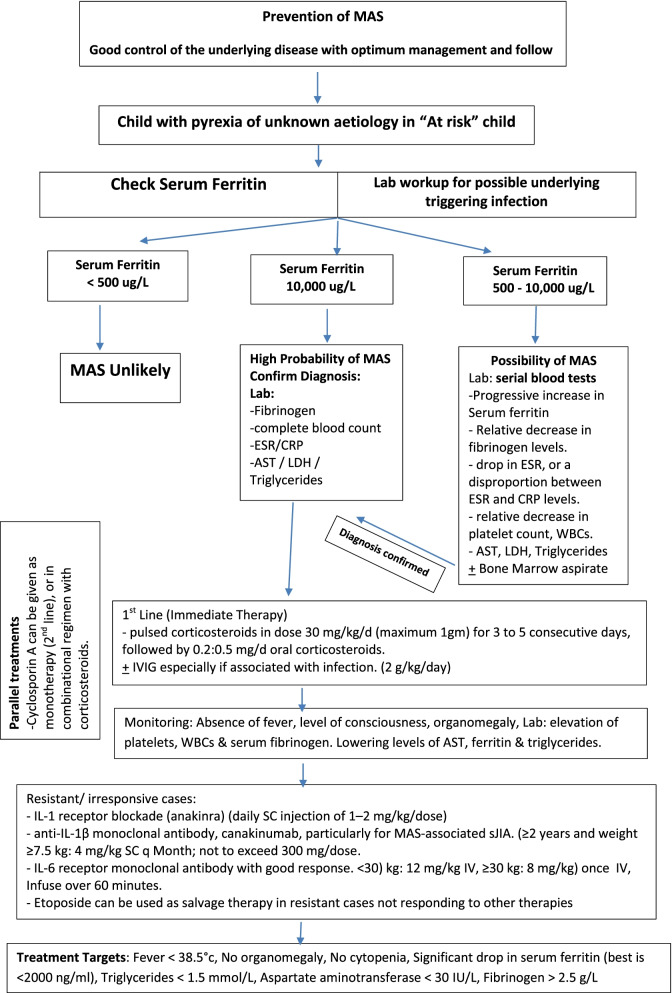
MAS management algorithm

Table 6 shows a summary of recommendations for MAS complicating sJIA, while Fig. 2 shows an algorithm for implementation of the MAS management approach in standard clinical practice.

**Table 6:** Summary of recommendations

No	Standard	Statement	LE	SoR	Mean rate ± SD	% of agreement	Level of agreement
	**Key points**	- Macrophage activation syndrome (MAS) is an acute, severe, and potentially lethal complication of several inflammatory diseases but seems particularly linked to both systemic juvenile idiopathic arthritis (sJIA) as well as in those with adult-onset Still disease. - MAS is classified among the secondary causes of hemophagocytic lymphohistiocytosis (sHLH). Other secondary HLH causes are infections and tumors and as a side effect of some drugs as aspirin, NSAIDs, etc. - MAS may be the first presentation of some patients with sJIA or lupus. - Several factors could trigger MAS incidence as a flare of the underlying disease and complicated infections. - Standardized diagnostic and treatment guidelines for MAS are currently lacking. - Up till now, there are no international guidelines or recommendations for the management of MAS.	1	A	8.39 ± 1.7	95.65	H
**Treat-to-target strategy**							
		*The target of therapy is to reach:* ▪ Fever < 38.5°C ▪ No organomegaly ▪ No cytopenia ▪ A significant drop in serum ferritin ( best is <2000 ng/mL) ▪ Triglycerides < 1.5 mmol/L ▪ Aspartate aminotransferase < 30 IU/L ▪ Fibrinogen > 2.5 g/L ▪ No hemophagocytosis in bone marrow (when feasible)	2	B	8.62 ± 1.6	95.65	H
**Management**							
1-a	**Prevention**	- MAS is not a preventable condition but lowering its incidence may be through good control of the underlying disease with optimum management and follow-up for laboratory markers. Poor disease control may be suggestive of MAS development. - Close follow-up of patients with inflammatory rheumatic diseases particularly those with past history of MAS, may help in preventing severe attacks.	2	B	8.52 ± 0.69	91.3	H
1-b	**First line of management**	- An early diagnosis and prompt aggressive initial treatment are both key factors for a favorable outcome. - The first line of treatment is parenteral administration of high doses or pulsed corticosteroids in dose 30 mg/kg/d (maximum 1g) for 3 to 5 consecutive days, followed by 0.2:0.5 mg/d oral corticosteroids.	1	A	8.26 ± 1.7	95.65	H
1-c	**Monitoring and follow-up (clinical and lab)**	**Clinically:** improving the overall status of the patient (e.g., conscious level) and absence of fever, improvement of clinical signs of systemic affection, petechial rashes as well as organomegaly. **Lab:** elevation of platelets, WBCs, and serum fibrinogen. Lowering levels of AST, ferritin, and triglycerides. **Assessment of inciting factors:** - Changes in the treatment regimen of JIA may be a provoking factor, so it is important to revise and reverse any recent alteration of medical therapy. - Infection may be a provoking factor; therefore, clinical manifestations suggestive of infection and positive cultures are helpful to confirm the diagnosis. - Neurological manifestations may require further investigations, e.g., MRA and MRV (to exclude vasculitis) and in some cases may require admission and proper monitoring in ICU.	3	B	8.13 ± 1.6	95.65	H
1-d	**Define resistant/irresponsive and severe cases of MAS**	- Resistant/irresponsive cases: cases refractory to conventional therapy with high-dose steroids. - Severely ill patients: patients with multiorgan failure.	1	A	8.26 ± 1.8	95.65	H
1-e	**Management of resisted and severe cases**	- In resisted cases: using of IVIG especially if associated with infection (2 g/kg/day, single continuous infusion). - For severely ill children: IL-1 receptor blockade (anakinra) (daily sc injection of 1–2 mg/kg/dose) has been remarkably effective when conventional therapy failed. - Human anti-IL-1β monoclonal antibody, canakinumab, has been reported to be successful in the treatment of MAS-associated sJIA (≥2 years and weight ≥7.5 kg: 4 mg/kg SC q month; not to exceed 300 mg/dose). - For severely resistant cases: (tocilizumab): recombinant, humanized IL-6 receptor monoclonal antibody with good response. <30) kg: 12 mg/kg IV, ≥30kg: 8 mg/kg) once IV infuse over 60 min. - Cyclosporin A was found to be effective in severe or corticosteroid-resistant MAS as IV or in an oral dose of 1.25 mg/kg PO BID (max 4mg/kg/d). - Cyclosporin A can be given as monotherapy, but in most patients, it is used as part of a combinational regimen with corticosteroids. - Etoposide can be used as salvage therapy in resisted cases not responding to other therapies - JAK inhibitors have promising experimental results for future expanded use in MAS management. - Anti-thymocyte globulin (ATG) can be used as salvage therapy for MAS but it is associated with high rates of infection; its safety and efficacy are not established in children.	3	B	8.56 ± 1.6	91.3	H
**MAS and COVID-19**							
2-a	**Key points**	- Coronavirus disease 2019 (COVID-19) in children is usually mild. However, in rare cases, children can be severely affected, and clinical manifestations may differ from adults. - Multisystem inflammatory syndrome in children (MIS-C) is an uncommon complication of COVID-19 that has a presentation similar to Kawasaki disease (KD), MAS, or toxic shock syndrome. - MIS-C should be suspected in a child with COVID-19 infection, particularly if presented with fever > 38 °C (100.4 °F) and at least two of the following suggestive clinical features: rash, gastrointestinal symptoms, edema of hands/feet, oral mucosa changes, conjunctivitis, lymphadenopathy, and neurologic symptoms. - The clinical presentation of MIS-C may include persistent fevers, gastrointestinal symptoms (abdominal pain, vomiting, and diarrhea), cardiovascular and respiratory affection, rash, and conjunctivitis. Patients typically present with 3 to 5 days of fever, followed by the development of shock and/or multisystem involvement. - Laboratory findings include lymphocytopenia, elevated inflammatory markers (C-reactive protein [CRP], erythrocyte sedimentation rate [ESR], D-dimer), and elevated cardiac markers (troponin, brain natriuretic peptide [BNP]). - MAS is one of the presentations of MISC with evidence of hemophagocytosis in the bone marrow.	4	D	8.28 ± 1.5	91.3	H
2-b	**Management strategy**	- Supportive care must be agreed with the experts who should take care of these patients, including pediatric ICU, pediatric infectious diseases, immunology, and rheumatology teams. - Vital signs, hydration, electrolytes, and metabolic status must be carefully monitored; fluid resuscitation, inotropic support, respiratory support, and, in rare cases, extracorporeal membrane oxygenation (ECMO) can be used in case of deterioration. - Patients with shock should be treated with volume expansion using Plasma-Lyte or Ringers lactate, vasoactive medications as epinephrine and norepinephrine are preferred. Use of antithrombotic therapy may be needed depending on the patient presentation and investigations (pediatric hematology consultation is advised in such cases). - Use of intravenous immunoglobulin (IVIG) 2g/kg, steroids, aspirin, and anticoagulation treatment is recommended at the same dosages that are usually administered to children with KD. - IL-1 receptor antagonist (anakinra), an IL-6 inhibitor (tocilizumab), and a chimeric IgG1κ monoclonal antibody specific for human TNFα (infliximab) could be used. - Whenever applicable, treat the associated bacterial infections.	4	D	8.24 ± 1.6	91.3	H

*LE* level of evidence according to the Oxford Centre for Evidence-Based Medicine (CEBM) criteria, *H* high level of agreement, *SoR* strength of recommendations, *T2T* treat to target, *COVID* coronavirus disease, *MAS* macrophage activation syndrome, *sJIA* systemic onset juvenile idiopathic arthritis, *sHLH* hemophagocytic lymphohistiocytosis, *IVIG* intravenous immunoglobulin, *ATG* anti-thymocyte globulin, *ECMO* extracorporeal membrane oxygenation, *ICU* intensive care unit, *CRP* C-reactive protein, *ESR* erythrocyte sedimentation rate, *sHLH* hemophagocytic lymphohistiocytosis, *KD* Kawasaki disease, *MRA* magnetic resonant arteriogram, *MRV* magnetic resonant venogram, *MIS-C* multisystem inflammatory syndrome in children, *COVID-19* coronavirus disease

## Discussion

Macrophage activation syndrome is a life-threatening condition in childhood, which is seen most commonly in sJIA, jSLE, Kawasaki disease, and infectious diseases. It is characterized by persistent unremitting fever, hepatosplenomegaly, lymphadenopathy, marked depression of all 3 blood cell components, intravascular coagulation, impaired liver function, and central nervous system dysfunction [5]. In a population research by M H Moradinejad et al. [20], the incidence of MAS was 4.2%, whereas the incidence of sJIA, SLE, juvenile idiopathic arthritis (JIA), and polyarticular RF negative JIA was 8.2%, 16.7%, and 2.8%, respectively, with a death rate of 40%.

The 2020 Egypt census revealed that the number of population is 104,124,440, of them 33.62% are children in the age range of 0–14 years old and 18.01% in the range of 15–24 years of age [18]. Though, up till now, there is no data on the prevalence of MAS in Egypt, having over 33 million children in Egypt would give a hint on the magnitude of the problem, even if estimated at the lowest prevalence rates.

Because MAS is a potentially fulminant disorder that can evolve into a life-threatening consequence of inflammatory rheumatic disease [19], it is critical to consider it in any juvenile rheumatic disorder characterized by rapid changes in general condition and a decrease in peripheral cells.

Expert consensus recommendations for the diagnosis and treatment of MAS are expected to play an essential role, given the absence of high-level evidence for diagnosis and management of MAS. The Delphi technique has shown to be a reliable tool for achieving such consensus and selecting future research directions [21]. The Delphi technique enlists the help of a group of experts to assess the level of agreement and settle disagreements on a topic [22]. The European League Against Rheumatism/American College of Rheumatology/Pediatric Rheumatology International Trials Organization (EULAR/ACR/PRINTO) Collaborative Initiative used a Delphi approach to define classification criteria for macrophage activation syndrome (MAS) in patients with sJIA. In agreement, this task was completed in a multistep process that included a Delphi survey and Web-based processes for determining expert consensus.

When using the Delphi process, consensus is reached when the percentage of people who agree or disagree is between 50 and 80% [21–23]. When the experts were asked about the possibilities of achieving a well-defined purpose in MAS, they came to a broad agreement. A total of 11 recommendations for diagnosis and 8 for treatment, and the percentage of agreement ranged between 86.9 and 95.7%, indicating a strong trend among the health care professionals to have a T2T approach for MAS management. A similar figure was reported in an earlier published work where an 82% consensus was achieved among 28 international experts [24].

At least one clinical criterion and at least two laboratory criteria must be present simultaneously for MAS to be diagnosed. Bone marrow aspiration is performed to search for evidence of macrophage hemophagocytosis, and it may be necessary only in doubtful cases [25]. These criteria were used to diagnose MAS in this study. Furthermore, the guidelines focused on early diagnostic techniques, with a focus on increasing increases in serum ferritin and a relative decrease in platelet count followed by a decrease in WBCs or fibrinogen levels, rather than an absolute decrease. Statements describing how to distinguish MAS from its other mimic illnesses were included to support the best diagnosis. MAS presentations in conjunction with disorders such as lupus, systemic auto-inflammation, and Kawasaki disease were also included.

Treat to target has established itself as a guiding strategy for the treatment of inflammatory arthritic conditions, and it is based on several principles: identifying a target and a tool to measure it, evaluating the target at a pre-determined time point, a commitment to change the therapy if the target is not met, and shared decision-making. Previous research has advocated either an evidence-based treatment method [26] or a therapy-based management algorithm [7]. In contrast, this guideline relied on treat-to-target strategy; the identified targets were to reach: fever < 38.5°C, no organometallic, no cytopenia, significant drop in serum ferritin (best is < 2000 ng/mL), triglycerides < 1.5 mmol/L, aspartate aminotransferase < 30 IU/L, and fibrinogen > 2.5 g/L. The guidelines discussed also preventive measures to lower the likelihood of developing MAS. A clear definition of resistant/irresponsive as well as severe cases has also been identified. Monitoring and follow-up parameters, both clinical and lab, were also identified and included in this work. Such a treat-to-target approach was reported to yield superior outcomes to standard treatment protocols, and several professional organizations have endorsed it as a fundamental therapeutic strategy [27]. To our knowledge, this is the first guideline to adopt such a strategy for the treatment of MAS.

Beyond the use of steroids which was highlighted in previous works [7, 8, 21], the algorithm proposed in this work for MAS management discussed the management of resistant and severe cases including the use of IVIG, biological treatment including IL-1 and IL-6 inhibitors, cyclosporine either mono or combined therapy. Also, the salvage role of etoposide and anti-thymocyte globulin and the promising experimental results of JAK inhibitors had been incorporated.

Given the current circumstances of the COVID-19 pandemic and possible serious conditions that appear to be linked to coronavirus disease 2019 in children [28], this guideline had a specific consideration for a rare but serious complication of COVID-19 known as a multisystem inflammatory syndrome in children (MIS-C). MIS-C resembles MAS in several manifestations; however, specific differences between both conditions have been identified. This work presented a discrete management strategy for the condition starting early diagnostic markers, clinical features, and tailored management protocol.

## Conclusion

This was an expert, consensus recommendation for the diagnosis and treatment of MAS, based on the best available evidence and expert opinion. The guidelines fill a gap in the literature as it presents a T2T approach for MAS. These suggestions should make it easier to find the best ways for diagnosing and management of this illness, as well as improve practice consistency, and promote the highest standards of care.

## Data Availability

The data will be available upon reasonable request.

## Abbreviations

ATG: Anti-thymocyte globulin
CEBM: Centre for Evidence-Based Medicine criteria
COVID: Coronavirus disease
CRP: C-reactive protein
ECMO: Extracorporeal membrane oxygenation
ESR: Erythrocyte sedimentation rate
H: High level of agreement
ICU: Intensive care unit
IVIG: Intravenous immunoglobulin
LE: Level of evidence
MAS: Macrophage activation syndrome
PICO: Population–Intervention–Comparator–Outcome
RCTs: Randomized controlled trials
sHLH: Hemophagocytic lymphohistiocytosis
sJIA: Systemic onset juvenile idiopathic arthritis
SLE: Systemic lupus erythematosus
SoR: Strength of recommendations
T2T: Treat to target
